# PhyloSuite v2: The development of an all‐in‐one, efficient and visualization‐oriented suite for molecular dating analysis and other advanced features

**DOI:** 10.1002/imt2.70095

**Published:** 2025-11-25

**Authors:** Dong Zhao, Tong Ye, Fangluan Gao, Ivan Jakovlić, Qiong La, Yindong Tong, Xiang Liu, Rui Song, Fei Liu, Zhong‐min Lian, Hong Zou, Wen‐Xiang Li, Gui‐Tang Wang, Benhe Zeng, Dong Zhang

**Affiliations:** ^1^ State Key Laboratory of Herbage Improvement and Grassland Agro‐Ecosystems, and College of Ecology, Lanzhou University Lanzhou 730000 China; ^2^ Key Laboratory of Biodiversity and Environment on the Qinghai‐Tibetan Plateau, Ministry of Education, School of Ecology and Environment Xizang University Lhasa 850032 China; ^3^ Institute of Fisheries Science Xizang Academy of Agriculture and Animal Husbandry Sciences Lhasa 850032 China; ^4^ Institute of Plant Virology Fujian Agriculture and Forestry University Fuzhou 350002 China; ^5^ School of Environmental Science and Engineering Tianjin University Tianjin 300072 China; ^6^ Hunan Fisheries Research Institute and Aquatic Products Seed Stock Station Changsha 410153 China; ^7^ State Key Laboratory of Breeding Biotechnology and Sustainable Aquaculture, Institute of Hydrobiology Chinese Academy of Sciences Wuhan 430072 China

**Keywords:** annotation, graphical user interface, MCMCtree, molecular dating, phylogenetic analysis, r8s, visualization

## Abstract

MCMCtree and r8s are among the most popular molecular dating tools in the current genomic era, but their utility is hampered by steep learning curves, particularly concerning input file formatting, the complexity of fossil calibration setup, tree visualization, and model selection. To enhance their usability and improve research efficiency, we developed three new tools: MDGUI (for molecular dating analysis), TimeTreeAnno (for timetree visualization), and MCMCTracer (for convergence assessment). We integrated these into the PhyloSuite v2 platform, along with MCMCtree and r8s plugins, to create a comprehensive molecular dating suite. Compared to existing solutions that we benchmarked, our toolkit offers a more intuitive interface and streamlined workflow, featuring visual calibration point configuration, support for multiple alignment formats, automated model selection and implementation for downstream analyses, one‐click pause/resume functionality, multithreading acceleration, and on‐demand MCMC convergence assessment and plotting. Furthermore, PhyloSuite v2 introduces other advanced features, including gene duplicate resolution during the extraction step, significantly accelerated data handling capabilities (specifically, format conversion and concatenation), deeper integration of the latest IQ‐TREE models and functions, and further streamlining of the entire phylogenetic analysis workflow. The update also includes adaptation to high‐resolution screens and numerous bug fixes. The source code for the new version of PhyloSuite is available at https://github.com/dongzhang0725/PhyloSuite.

## Highlights

- PhyloSuite v2 integrates a unified molecular dating analysis suite that seamlessly connects data processing, parameter configuration, molecular dating analysis, and results visualization within a streamlined workflow.
- Supports drag‐and‐drop operation, automatic import of upstream results, and intuitive fossil calibration visualization for efficient and user‐friendly operation.
- Compatible with various common alignment file formats and fully supports all major evolutionary models.
- Offers multithreaded acceleration and pause/continue options, achieving markedly enhanced efficiency and usability compared with command‐line MCMCtree.
- Incorporates comprehensive downstream analyses, including real‐time MCMC convergence monitoring, MCMC statistical summaries and plotting, and timetree visualization and annotation.

## Graphical abstract

This graphical abstract illustrates the integrated molecular dating workflow in PhyloSuite v2. The new Molecular Dating Suite unifies data configuration, analysis, and visualization into a single, streamlined process. It features an intuitive interface with drag‐and‐drop operation and visual fossil calibration on tree nodes, supports broad alignment formats and complex models, and executes analyses efficiently with multithreading and pause/continue functionality. The final stage of the workflow provides enhanced visualization tools, enabling MCMC convergence assessment with MCMCTracer and the generation of time‐scaled trees with TimeTreeAnno.

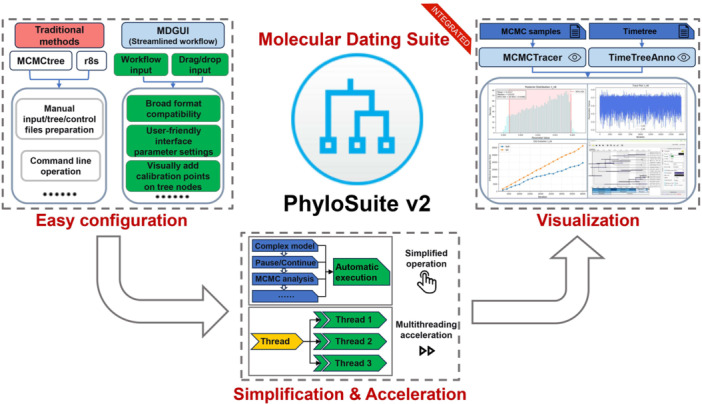

## INTRODUCTION

Phylogeny, the study of evolutionary and genetic relationships and common ancestry among species, provides a crucial framework for investigating fundamental biological questions, such as patterns of divergence, speciation, evolution of ecological strategies, demographic changes, and migration patterns of species [1]. Along with fossil data, molecular dating is critical to this framework, as it allows researchers to temporally calibrate these evolutionary events, thus transforming a simple tree of relationships into a dynamic historical narrative [2]. The development of Bayesian methods popularized molecular dating studies. These advanced methods allow algorithmic incorporation of complex aspects of molecular evolution and fossil information—typically as node priors—to calibrate estimated divergence times from modelled changes in DNA or amino acids [3, 4]. As the Bayesian method is computationally demanding, there are also alternative, faster, methods for estimating divergence times, such as the penalized likelihood (PL), and nonparametric rate smoothing (NPRS) [5, 6]. These methods enable researchers to quantify evolutionary timescales through DNA or protein sequence analysis, with each method offering distinct advantages in handling clock model violations and fossil calibration uncertainties [7]. Currently, the most popular tools for molecular dating analyses comprise MCMCtree, BEAST, and r8s.

MCMCtree, a program incorporated into the PAML software package, employs Bayesian inference to conduct molecular dating [8, 9]. MCMCtree can use genome‐scale datasets and relies on Bayesian inference to obtain divergence times [10]. The program is operated via the control file, which includes settings such as likelihood calculation options, molecular clock model selection, loose constraints on root age, and so forth. [11]. Currently, there are two ways to use MCMCtree: via the command prompt (CMD) and via the official graphical interface (GUI) application, pamlX [12]. The first option requires a certain level of proficiency in CMD operation and familiarity with the Linux system, thus diminishing its user‐friendliness. In pamlX, file input and parameter settings for MCMCtree steps can be done through the GUI, with annotations for each parameter displayed in the option boxes. However, pamlX suffers from several limitations that hinder its usability and efficiency, including: (i) the lack of integrated timetree visualization; (ii) a text‐based system for managing fossil calibrations, which many users may find time‐consuming and difficult to navigate; (iii) incomplete implementation of substitution models—MCMCtree comprises only five basic models, so the use of other models requires manual editing of control files (e.g., missing models comprise WAG, MtZoa, LG, etc.); (iv) no option to preview or modify control files; (v) computational inefficiencies due to a lack of a multithreading, making large‐scale datasets analyses slow; and (vi) the lack of an option to easily pause/resume analyses (e.g., users are required to manually modify the control file when resuming an interrupted analysis) (Table 1). These factors not only make the learning curve steeper, but also slow down the analysis of large‐scale genomic datasets, requiring considerable time investment even from experienced users during practical operations. There are two tools designed to address some of the shortcomings listed above: (1) MCMCtreeR is an R language package designed for preparing time priors for MCMCtree analysis and plotting time‐scaled phylogenies [13] and (2) TVBOT can be used to visualize timetrees resulting from molecular dating analyses [14]. However, neither of these two tools can be used to conduct the molecular dating analysis, so it is still necessary to operate multiple software programs and transfer files between them to conduct a complete analysis.

**Table 1:** Functionality comparison of PhyloSuite and other molecular dating tools.

Function	pamlX	MCMCtreeR	BEAST2	r8s/pyr8s	TVBOT	MDGUI
Drag‐drop functionality	√	×	√	×	×	√
Workflow import of upstream results	×	×	×	×	×	√
Support for multiple alignment formats (FASTA, NEXUS, PHYLIP, CLUSTAL, and etc.)	×	n/a	×	×	n/a	√
Automatic model selection followed by molecular‐dating analysis	×	n/a	×	×	n/a	√
Visually adding the calibration to the desired tree node in the phylogenetic tree interface	×	×	×	×	×	√
Integrating IQ‐TREE for Hessian matrix calculation, supporting more models than standard MCMCtree	×	n/a	n/a	n/a	n/a	√
One‐click molecular dating pipeline	×	n/a	×	×	n/a	√
Multithreading to accelerate the molecular dating analysis	×	n/a	√	×	n/a	√
Pause/continue the analysis, summarize the results and generate the timetree at any point	×	n/a	√	×	n/a	√
Molecular dating tree visualization and annotation	×	√	×	×	√	√
Tracer‐like summarization and visualization	×	×	√	×	×	√
One‐click interactive MCMC results visualization with real‐time convergence assessment, with no extra tools needed	×	n/a	×	n/a	n/a	√

Abbreviation: n/a, not available.

BEAST also employs Bayesian inference to perform a range of evolutionary analyses, comprising the molecular dating [15]. It provides a graphical user interface with comprehensive functionality and includes additional tools, such as Tracer. However, it does not offer features such as automatic adjustment of input file formats, automatic prediction of optimal models, visual management of fossil calibrations directly on the tree interface, one‐click summarization of results whenever needed, or direct visualization and annotation of timetrees.

Finally, r8s employs PL and NPRS methodology to perform molecular dating [16]. However, it lacks a graphical user interface (GUI), so users are required to manually write and format a command file according to analysis requirements, including the tree file information, parameter configuration, and calibration settings. Detailed documentation is provided for each parameter, but the process can be time‐consuming and prone to formatting errors. It should be noted that a GUI for r8s is available through the Python package integration (pyr8s), it has limited functionality [17]. Moreover, r8s was developed only for Linux and macOS systems, so its deployment on Windows comprises multiple hurdles for most users.

**Figure 1:**
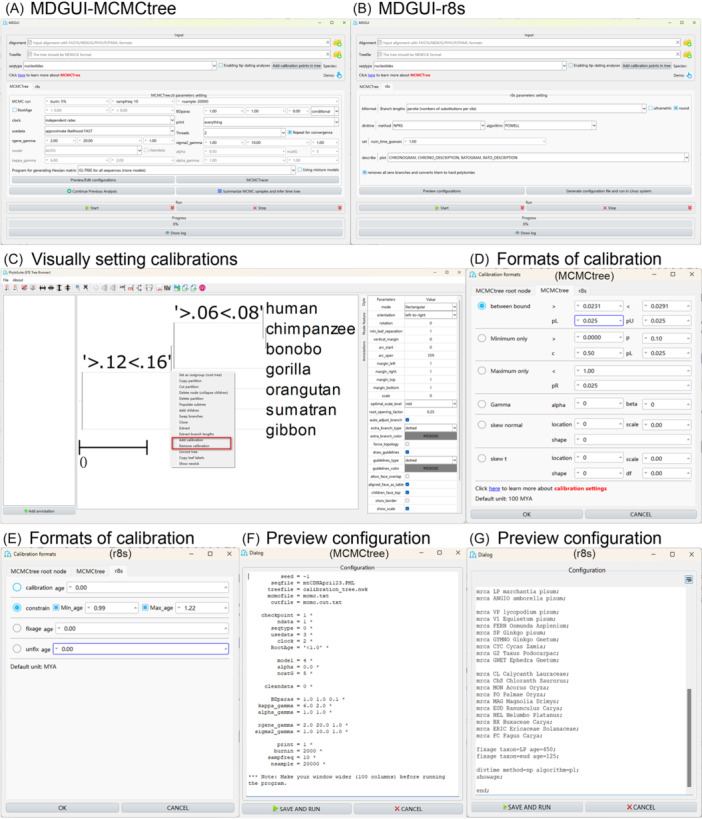
The interface and main functions of the molecular dating suite—MDGUI—in PhyloSuite. (A) The MCMCtree interface of MDGUI. (B) The r8s interface of MDGUI. (C) Visually setting and managing calibration points on the tree. (D) Selecting different types of MCMCtree tree file calibration styles. (E) Formats for r8s tree file calibration. (F) A preview of the control file contents of MCMCtree. (G) A preview of the control file contents of r8s.

To address the shortcomings listed herein, we developed MDGUI, a graphical interface for molecular dating that integrates MCMCtree and r8s plugins, while also incorporating TimeTreeAnno for timetree annotation and MCMCTracer for convergence assessment. We incorporated these tools into the new version (v2) of our platform, PhyloSuite [18, 19], to create a comprehensive molecular dating suite. Beyond incorporating the existing features of MCMCtree via pamlX, we significantly expanded the functional framework with practical features, including streamlined input‐output processes, tree file visualization based on the ETE package [20], and visual management of fossil calibration information to ensure accurate placement of calibration points. The suite also provides a flexible analytical control through options to pause and resume analyses at will, without the need to manually edit the control file, a rerun function for convergence assessment, and the capability to summarize MCMC results and generate timetree after an interruption (Figure 1). Furthermore, we implemented multithreading functionality to enable simultaneous MCMC runs, greatly improving the efficiency for large datasets. Benchmarking on the published data set confirms that molecular dating analyses complete significantly faster with multithreading acceleration, based on a comparison of runs with an equal number of MCMC samples (Figure S1) , while the results show minimal differences of node time estimates between PhyloSuite v2 (4 threads) and command‐line MCMCtree (Figure S2). MDGUI simplifies several complex processes into one‐click operations, such as selecting the best‐fit model and directly applying it in downstream molecular dating analysis. We also provide full integration of r8s into the interface, providing convenient functionalities for parameter configuration, command file generation, and analysis execution. MDGUI leverages these functionalities to enable visual setting of time calibrations on the tree, automatically generating corresponding commands (e.g., MRCA, CONSTRAIN, FIXAGE). After parameter configuration, users can choose to either generate the required input files for r8s or run the analysis directly.

**Figure 2:**
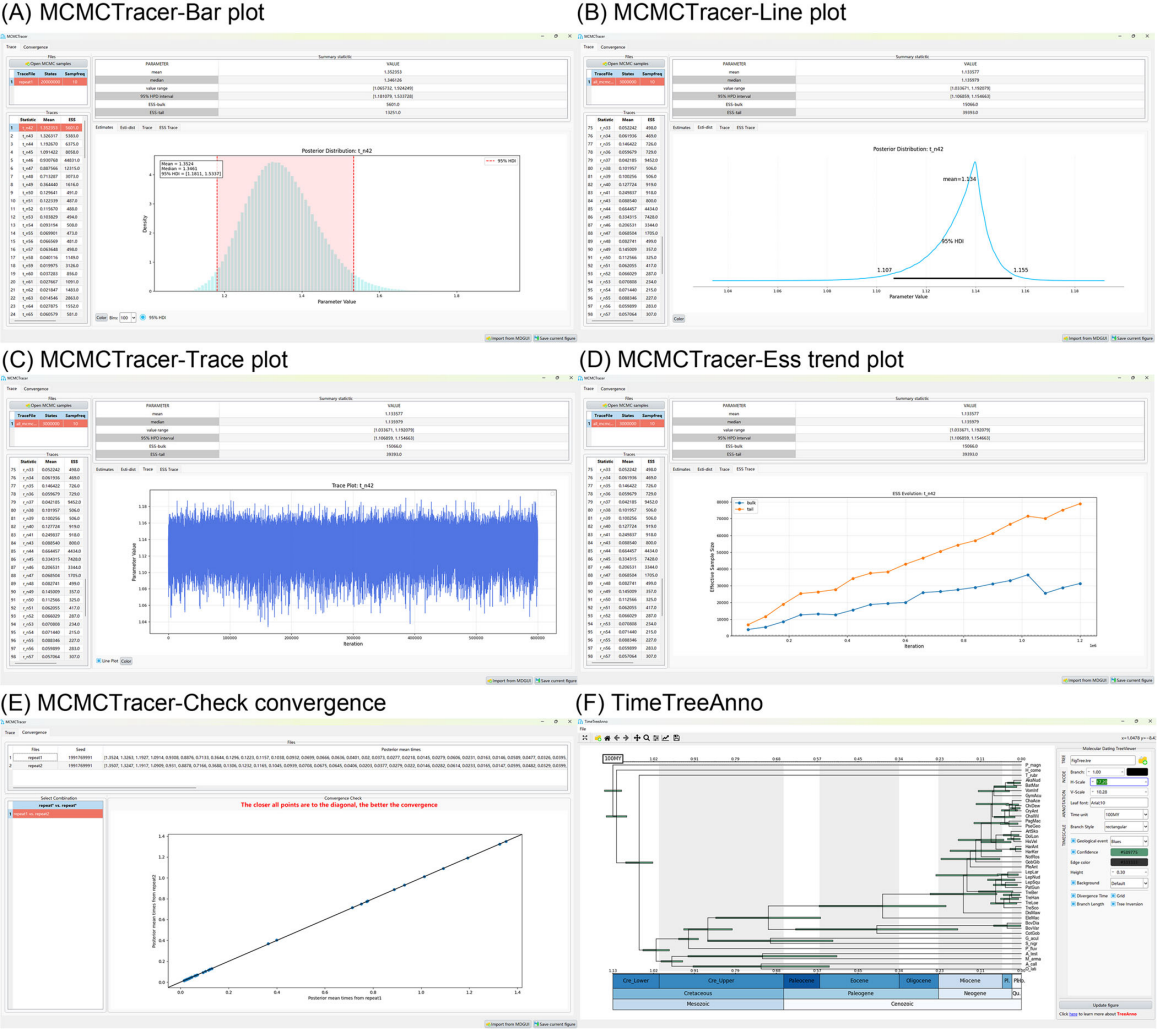
The interface of MCMCTracer and TimeTreeAnno. (A−C) Different types of graphics to visualize MCMC samples. (D) Visualization of ESS trends. (E) Convergence diagnostics. (F) Timetree visualization and annotation customization.

Regarding the other two newly designed tools, TimeTreeAnno enables sophisticated visualization of molecular dating trees, allowing users to customize annotations and export images in various formats. MCMCTracer provides a comprehensive summarization of MCMC results, similar to Tracer [21], including the ESS (Effective Sample Size), confidence interval estimates, median values, and other key statistics. Beyond the Tracer's capabilities, MCMCTracer offers an expanded functional repertoire—comprising scatter, histogram, box, and line plots—for visualizing MCMC samples. This enhanced toolkit allows for a more thorough assessment of analysis reliability. It can also visually compare two MCMCtree analyses to evaluate convergence (Figure 2). In this way, the molecular dating suite in PhyloSuite and its plugins offer users a more comprehensive functionality than existing tools we benchmarked (Table 1).

The PhyloSuite v2 also introduces advanced features across the entire platform, including accelerated file handling and format conversion, sophisticated resolution of duplicated genes during sequence extraction, deeper integration of the latest IQ‐TREE models and functions, and a refined adaptive interface for optimal display on high‐resolution screens. In addition, a series of bugs have been resolved, such as the progress bar issue in ModelFinder, plotting errors in sequence saturation analysis, and problems with IQ‐TREE failing to automatically recognize the latest models in ModelFinder. By consolidating these functionalities into a single, user‐friendly platform, we have solidified the position of PhyloSuite as an integrated and powerful suite for molecular data analyses. It streamlines a range of evolutionary analyses, significantly reducing technical barriers and manual effort, allowing researchers to focus on biological interpretation rather than computational execution.

## IMPLEMENTATION

### File input optimization

MDGUI, coded in Python and incorporated into the latest PhyloSuite (v2), is designed to provide a streamlined and user‐friendly experience while retaining the full functionality of both MCMCtree and r8s (Figure 1A,B). Unlike pamlX, which offers a single‐file input method in the MCMCtree module, MDGUI provides three flexible options: (1) automatic input of upstream analysis results (workflow input), (2) drag‐and‐drop import, and (3) via the import button. In workflow mode, MDGUI automatically detects result files from upstream analyses, such as sequence alignment files and phylogenetic tree files from the results of IQ‐TREE, MrBayes, or fasttree plugins implemented in PhyloSuite. Regardless of the input method, the program automatically validates and converts the file formats as needed. For example, sequence alignments in FASTA, NEXUS, and PHYLIP formats are automatically converted to the PML format required by MCMCtree. In addition, MDGUI also verifies the correspondence of species names between sequence files and tree files, and displays the species count. For tree input, MDGUI also provides a visual representation of the phylogram. Users can select different annotation formats (for details, see MCMCtree user guide) to visually assign fossil calibrations directly to tree nodes (Figure 1C,D). As root node calibration is a mandatory step in MCMCtree, MDGUI enforces this requirement by automatically launching the configuration window if the parameter was not set before, guiding users through the calibration process.

Within the r8s module, users can similarly import a tree file and visually add calibration information. The interface supports four command formats: “calibrate,” “constrain,” “fixage,” and “unfixage” (Figure 1E). Based on the annotated tree and user‐defined parameters, the program automatically generates the corresponding commands. Users can review and modify these commands through the “Preview/Edit configuration” interface before running the analysis with updated parameters (Figure 1F,G).

### Execution process optimization

MCMCtree natively supports only five nucleotide substitution models (JC69, K80, F81, F84, HKY85) for direct analysis, so implementing advanced nucleotide (e.g., T92, T93, GTR, UNREST) or amino acid models (e.g., WAG, MtZoa, LG) requires configuring the “usedata” parameter for approximate likelihood calculation alongside Baseml/Codeml programs—a process demanding complex manual procedures. For protein models, this involves seven sequential steps: setting “usedata = 3” in the control file and executing MCMCtree; modifying the generated “tmp*.ctl” by specifying the best‐fit model in “aaRatefile,” appending “+F” to the model parameter where applicable, and configuring “+G” in “ncatG” and “alpha” if needed; running “Codeml” to produce the Hessian matrix (rst2); renaming rst2 to “in.BV”; and finally setting “usedata = 2 in.BV” in the main control file for divergence time estimation. Nucleotide model implementation, though simpler (typically three steps), still necessitates manual intervention as detailed in the MCMCtree tutorial [22].

To simplify this complex workflow, we optimized it into a single‐click operation where MDGUI automatically handles all intricate manual steps in the background and presents results directly to users. This enhancement provides all available models in the MDGUI interface, grouped by sequence type (nucleotides and amino acids), enabling users to select any non‐standard models (such as the GTR, UNREST, or 18 amino acid models). Additionally, we also added the “AUTO” option to address situations where users are uncertain which substitution model is the best fit to the data. After selecting this option, the program will invoke ModelFinder [23] plug‐in to calculate the best‐fit model for the data set, and then use MCMCtree and the optimal model to conduct molecular dating analysis. Regarding the Hessian matrix calculation, in addition to the Baseml and Codeml tools provided by PAML, we have also integrated IQ‐TREE for Hessian matrix computation, which supports more models [24].

MDGUI offers a clear strategy for parameter configuration in MCMCtree, exposing commonly modified parameters while intelligently automating or setting defaults for others. For example, parameters that can be reliably determined from the input data are set automatically (e.g., data type). If a user inputs amino acid data but mistakenly selects a model designed for nucleotide sequences, MDGUI will display a pop‐up window to remind them to choose an appropriate amino acid model. For parameters that are complex, yet crucial, we have redesigned their presentation. A key example is the usedata parameter, for which we have consolidated the options “2: normal approximation” and “3: out.BV” into a single, simplified, and default option labeled “approximate likelihood FAST”, which streamlines the workflow for most users. When users opt for non‐standard nucleotide or amino acid models, the “usedata” parameter automatically switches to “approximate likelihood FAST”, simplifying the setup process and ensuring that correct analysis parameters are applied without manual intervention. The clock model configuration has been kept as in MCMCtree, and the “independent rates” parameter is set as the default option in the interface. For fossil calibration bounds, a default soft boundary of 2.5% is applied in the MCMCtree calibration format interface, allowing a small probability for the bounds to be violated. The advanced parameters, such as “BD paras,” “rgene gamma,” and others, are set to defaults suited for the example datasets for this version. For r8s analyses, the MDGUI provides an intuitive interface that maps directly to the parameters described in the official pyr8s and r8s documentation. This allows users to configure their analyses with guidance from the standard references. Furthermore, if users need to add parameters not provided in the MDGUI interface, they can click the “Preview/Edit configuration” button to make changes via the interactive editing interface (Figure 1F,G).

While the “approximating the likelihood” mode implemented in MCMCtree accelerates genomic data analysis, it may not fully meet the demands of large‐scale data sets. To address this, we incorporated a multithreading acceleration feature. Users can specify the number of threads via the “Threads” parameter in the MDGUI's interface. If the number is greater than one, the program will execute multiple parallel MCMC chains, with identical settings but with different “seed” values, drastically reducing the time required for the analysis.

The PhyloSuite v2 itself uses approximately 30−350 MB during operation, with minimal CPU utilization (e.g., approximately 0.05% observed on an Intel Ultra 5 125H processor). As our optimizations were focused on workflow efficiency and parallelization, they did not alter the fundamental resource consumption of incorporated algorithms, so the computational demands for MCMCtree and r8s are consistent with their standalone versions. Therefore, we recommend users consult the official PAML and r8s documentation for hardware requirements.

For convergence assessment of MCMCtree analysis, the official tutorial recommends repeating the analysis with the same configurations but different seeds. This generally requires users to manually run the analysis at least twice, making the process time‐consuming and potentially cumbersome. To optimize this procedure, streamline the convergence assessment process (see below), and allow users to quickly assess the reproducibility of their results, we introduced a “Repeat for convergence” checkbox. Checking this box automatically repeats the analysis with no additional user input required. This function can also be combined with the multi‐threading. When users select both “Repeat for convergence” and a specific number of threads, MDGUI will split the analyses into two repeats according to the thread number. For instance, selecting four threads allocates two threads to each repeat. If an odd number of threads is selected, MDGUI reduces the core count by one (e.g., if 3 threads are selected, it will use 2 cores) and then evenly distributes the remaining threads between the two repeats. Notably, even if a single thread is selected, MDGUI automatically utilizes two cores to complete the two repeated analyses, allowing efficient convergence assessment.

Beyond the parameter configuration and execution, we also added a convenient control file preview feature. After setting all interface parameters, users can click the “Start” button to run the MCMCtree program directly. button launches an interactive editing interface. Here, the auto‐generated configuration is displayed in a modifiable text format. This powerful mode allows users to not only adjust pre‐set values but also to insert new parameters, including those not directly exposed in the MDGUI interface, ensuring precise configuration. Once modifications are complete, users can simply click “SAVE AND RUN” to initiate the analysis.

As for r8s, we incorporated different versions, depending on the operating system: the original r8s for Linux and macOS platforms, and the pyr8s package (https://github.com/iTaxoTools/pyr8s) for Windows platforms. Due to inherent differences between these implementations, we provided distinct interfaces for each operating system. Primary distinctions lie in parameter configuration: the Linux and macOS versions offer additional options such as “cvnum,” “shape,” and “smoothing,” allowing for more flexible parameter settings. After configuring the parameters via the graphical interface, users can initiate analysis directly or preview and modify the command file before execution.

Beyond its role in calculating the Hessian matrix for MDGUI, the integration of IQ‐TREE has been substantially enhanced in PhyloSuite v2. In addition to previous features, the current implementation supports the latest IQ‐TREE models, including the empirical nucleotide models “NQ.bird,” “NQ.insect,” “NQ.mammal,” and various mixture models. Furthermore, key functions, such as alignment trimming and the “fast search” option (designed to resemble the speed of FastTree) [25], have been seamlessly integrated into the relevant workflow steps. Underlying improvements include the resolution of a progress bar bug in the ModelFinder component and successful incorporation of the MixtureFinder functionality [26], significantly enhancing both the stability and model selection capabilities within the suite.

### Timetree visualization and annotation

Beyond the design and functional optimization of MDGUI, we have also developed a dedicated tree visualization and annotation tool, TimeTreeAnno, built on the matplotlib [27] and phytreeviz (https://github.com/moshi4/phyTreeViz) packages in Python. This tool can display and annotate the timetrees generated by MCMCtree, BEAST, or r8s, and it includes several adjustable features for visualization of confidence intervals for node ages, displaying corresponding geological timescales, and adding cluster annotation. The interface also provides various customizable display settings, such as background color options, auxiliary time axes, and the ability to reverse tree orientation (Figure 2F). These options allow users to personalize the visualization of the timetree according to their preferences. It also offers a selection of different theme colors, and supports exporting and saving the trees in jpg, png, svg, and pdf formats.

### Real‐time convergence checking and statistics plots

To improve analytical flexibility, MDGUI allows users to pause an ongoing analysis at any time, summarize the current MCMC results and generate timetree. Users can also perform convergence diagnostics on‐the‐fly while the program is running. These interim outputs can be seamlessly imported into MCMCTracer, whether the analysis is in progress or completed. MCMCTracer is a dedicated real‐time diagnostic module built upon the powerful matplotlib and ArviZ libraries [28]. It enables one‐click dynamic monitoring of analysis progress during the MCMC execution, including the scatter plot of posterior mean times, as well as the statistics and plots of Effective Sample Size (ESS), 95% highest posterior density (HPD) intervals, etc (Figure 2A‐D). The scatter plot of posterior mean times from replicate analyses (e.g., repeat1 and repeat2) is used as the key diagnostic criterion for assessing convergence (Figure 2E). When chains have converged to the same posterior distribution, the data points in a scatter plot of these values are expected to cluster closely around the x = y line. In terms of visualization capabilities, MCMCTracer is different from Tracer by offering new ESS trend plots (which display the growth trajectory of effective sample size). Furthermore, MCMCTracer supports exporting plots with customizable resolution (DPI) and in various file formats, including JPEG, PNG, and PDF.

**Figure 3:**
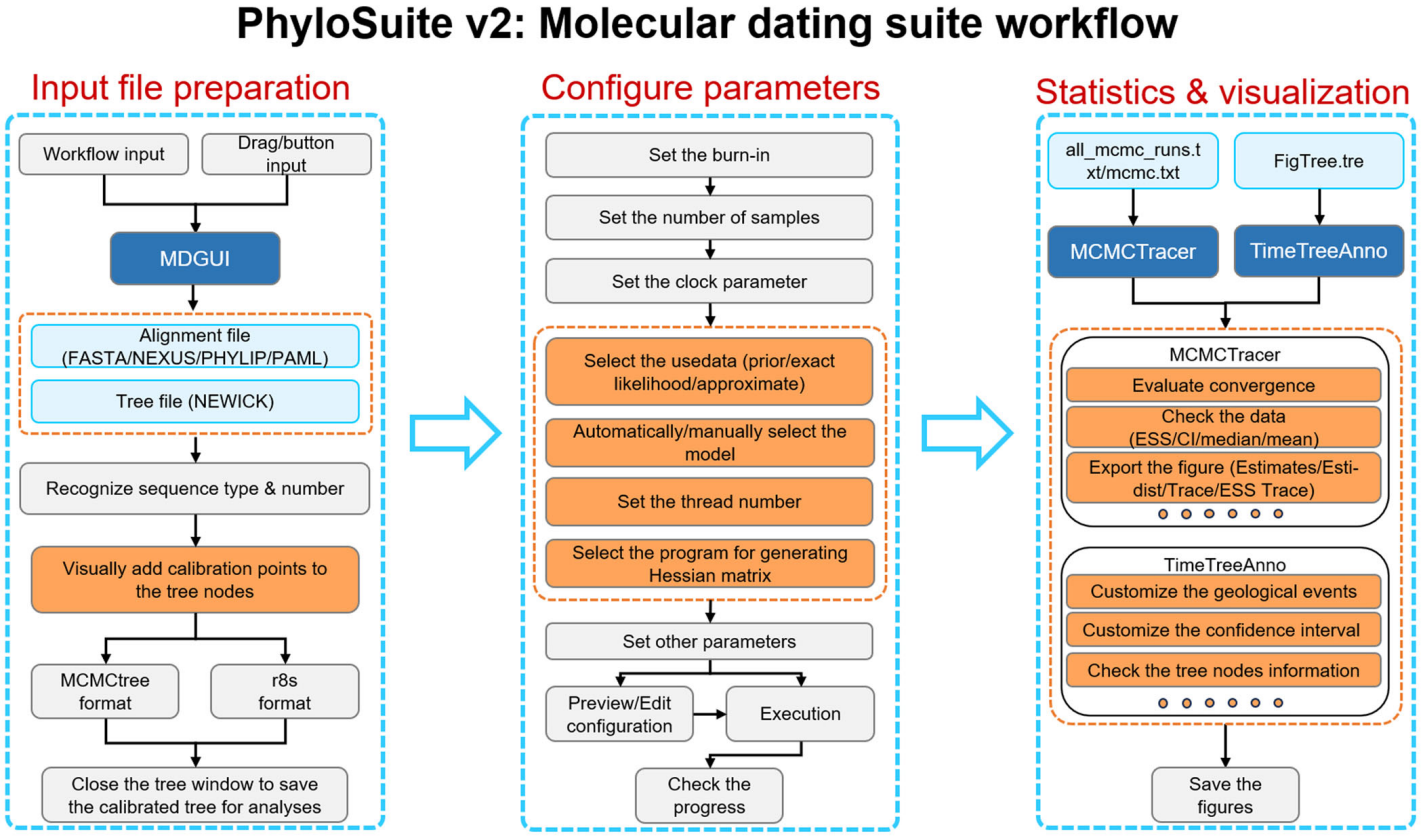
The workflow of the molecular dating suite in PhyloSuite v2.

Collectively, MDGUI streamlines the entire molecular dating workflow (Figure 3), from input preparation to analysis and visualization through intuitive automation. By transforming complex procedures into single‐click operations, resolving parameter conflicts intelligently, and accelerating computations via parallelization, the tool significantly reduces manual effort while ensuring methodological rigor. This integrated approach empowers researchers to focus on biological interpretation rather than technical execution, ultimately advancing efficiency and reproducibility in divergence time estimation.

### User interface and functional improvements

In addition to molecular dating–specific functions, PhyloSuite v2 has been strengthened with advanced capabilities for general phylogenetic data processing. These enhancements include a substantially faster file format conversion engine, an optimized sequence concatenation function with optional codon position partitioning, and an improved gene extraction module that can effectively resolve gene duplicates (Figure S3). The latter function relies on sequence comparisons with the closest related species and the detection of internal stop codons within CDS sequences. The graphical interface has been refined for improved adaptability across different screen resolutions, ensuring optimal display on high‐resolution screens. Furthermore, the post‐analysis summarization for MrBayes analysis has been optimized for greater speed.

## APPLICATION EXAMPLE

### Import files

To illustrate the usage protocol, we utilized the tree and sequence files from the PhyloSuite examples (http://phylosuite.jushengwu.com/example.zip). The analysis begins by accessing the MDGUI interface via the “Phylogeny” drop‐down menu. Users should first locate the input files on their local computer. The tree and the alignment files (example files in the “example” > “MCMCtree” folder) can then be dragged and dropped into the respective input boxes (Figure S4). Upon successful loading, the interface automatically displays the number of species and the identified sequence type (“nucleotides” in this case). Notably, the program features a built‐in format conversion function that automatically transforms common sequence file formats (FASTA, NEXUS, PHYLIP, etc.) into the required PML format, thereby preventing potential errors caused by improper file formats.

For fossil calibration, users should click the “Add calibration points in tree” button to activate the tree visualization interface. When processing a tree file containing pre‐existing fossil time annotations, the program automatically recognizes and displays these constraints. To add new calibrations, users can right‐click on the target node (Figure S5), select “Add calibration,” choose the desired annotation format, and set the time parameter (unit: 100 million years). The new calibration will immediately appear on the designated branch. We provide an example of a tree that contains a complete annotation in (Figure S6). To modify existing fossil constraints, users can right‐click the annotated node, select the “Remove calibration” option to delete the existing annotation, and then add updated fossil information as described above. It is important to emphasize that MCMCtree algorithm requires that fossil calibration is specified for the root node of the input tree. To facilitate this, we have designed the “MCMCtree root node” panel within the “Calibration formats” window. If users attempt to close the window without calibrating the root node, the program will pop up a warning window and automatically reopen the interface. For r8s, the operation is similar, aside from relying on different calibration formats and interface design. Notably, r8s does not require a root node calibration, so this step is also optional in PhyloSuite.

### Parameter settings

After file input, users can configure analysis parameters through the intuitive interface. All adjustable parameters correspond to those described in the control file documentation (http://abacus.gene.ucl.ac.uk/software/MCMCtree.Tutorials.pdf). Notably, users no longer need to manually set the seed value, as the program will automatically generate random seed values, while preventing duplicates during multithreading acceleration. For the core analysis, setting the “usedata” parameter to “approximate likelihood FAST” enables the approximating likelihood calculation followed by MCMC analysis. The modeling configuration offers flexible options; users may manually select substitution models (e.g., JC69, or some other) or choose the “AUTO” option to automatically invoke ModelFinder to calculate the best‐fit model for the data set, which MDGUI then automatically uses for the molecular dating analysis. The evolutionary clock model provides three distinct options: (1) “global clock” (constant rate model), (2) “independent rates” (independent rate model), and (3) “correlated rates” (autocorrelated rate model). For our example, we selected the “independent rates” model and configured associated parameters, including the birth‐death process (BDparas), substitution model (kappa_gamma and alpha_gamma), and the average replacement rate (rgene_gamma) (Figure S4). If the “model” parameter is set to “AUTO,” the program updates these parameters automatically. Before execution, users can review all configured parameters by clicking “Preview/Edit configuration,” which generates and displays a complete control file for verification. For other parameter settings, see comprehensive MCMCtree tutorials (https://github.com/abacus-gene/paml/wiki/MCMCtree).

In the r8s example, we set the “length” parameter to “persite,” select the “NPRS” method and the “POWELL” algorithm, set “num_time_guess” to “1,” and customized the “describe” parameter. Detailed explanations of all parameters are available in the r8s user guide (https://naturalis.github.io/mebioda/doc/week1/w1d5/r8s1.7.manual.pdf). After configuring the parameters, clicking the “Start” button will begin the r8s analysis.

### Running the analyses and checking the results

After configuring all MCMCtree parameters, users can initiate the analysis by clicking the “Start” button. The program then executes the molecular dating analysis based on the imported files and run settings. The interface automatically displays a “Log” window, providing real‐time progress tracking. By default, the program generates a results folder timestamped with the analysis start time, unless users explicitly designate an alternative output folder name. For enhanced computational efficiency, the program supports multithreading. When enabled, the program will run multiple MCMC chains simultaneously, each generating a separate “run*” folder within the default results directory. If the “Repeat for convergence” option is also enabled, “run*” folders will be located within the “repeat1” and “repeat2” subfolders, containing the results of two separate analyses. Upon completion, the MCMC samples from each repeat are merged into a single file for MCMC summarization and timetree generation, significantly speeding up the overall analysis.

During analysis, users can click the “Summarize MCMC samples and infer time tree” button to summarize samples and infer the timetree, or click “MCMCTracer” button to assess the convergence, all without stopping the MCMCtree program. To halt an MCMCtree run while simultaneously summarizing samples, click on the “Stop” dropdown menu and select “Stop the run and infer the time tree”. This stops the analysis, summarizes the current MCMC samples, and generates the timetree file. MCMCtree automatically saves the chain state to “mcmctree.ckpt” file. If convergence has not been reached, users can resume the analysis (from the interruption point) by selecting “Continue Previous Analysis” and choosing the corresponding output folder.

After the analysis is complete, a pop‐up window will appear, allowing the user to choose whether to import the results into MCMCTracer or TimeTreeAnno for further analysis (workflow input, see the following two sections).

After r8s analysis completes, aside from the standard result file (including “age_rates.csv”), additional files are also created based on the options selected in the “describe” parameter. Among them, “chronogram.nwk” represents the timetree.

### MCMCTracer analysis

MCMCTracer is an integrated analysis tool designed for summarizing and visualizing MCMCtree output files, providing functionality similar to Tracer. It operates on the MCMC samples generated by MCMC analyses, performing comprehensive statistical evaluations and graphical representations. Users can access the MCMCTracer interface either through the workflow input from MDGUI results or by manually importing the results using the “Import from MDGUI” button. The intuitive interface has two tabs: (1) "Trace" provides Tracer‐like statistics and plotting (Figure S7), (2) "Convergence" provides visualization of posterior mean values to assess convergence (Figure S8). After the data are imported, MCMCTracer will calculate statistical values (e.g., sampling frequency and ESS) and visually displays results via histograms, line plots, box plots, and scatter plots. Users can also modify parameters like colors or bins, and save all generated figures. For convergence assessment, see the previous section.

When the “Repeat for convergence” option is enabled, MCMCTracer directly processes samples from the automatically generated “repeat1” and “repeat2” folders. If not enabled, users can either manually perform a second analysis and import both folders, or—for multiple‐thread analyses—import the single results folder for automatic pairwise convergence assessment for different runs. This pairwise approach is also applied when more than two results or runs are imported, ensuring a comprehensive evaluation of convergence across all available data.

### Molecular dating tree visualization

Aside from the automatic workflow input of the timetree from MDGUI results, users can also open the TimeTreeAnno interface either by directly double‐clicking the “FigTree.tre” file from MCMCtree results or "chronogram.nwk” file from r8s results, or alternatively via the “Phylogeny” menu bar in the main PhyloSuite interface. Here, users can display confidence intervals, clusters, geological events, and other graphic elements on the tree diagram. We provide extensive customization options for these elements, including: phylogenetic tree time units, background color, geological event color themes, confidence interval colors, and per‐element display controls (Figure S9), offering significant flexibility in tree visualization.

### Limitations

The current version of PhyloSuite v2 has certain limitations to acknowledge. The current implementation of MDGUI does not support partitioned sequence analysis. The program automatically converts input alignments into a non‐partitioned PML format, irrespective of the original partitioning scheme. This uniform treatment may reduce model accuracy for datasets with heterogeneous evolutionary patterns across partitions. However, if users select IQ‐TREE for Hessian matrix calculation, its mixture models can be used to mitigate the impact of data heterogeneity. Support for partition analysis is planned for a future update. Additionally, if users encounter issues during use, they can consult the troubleshooting guidelines (Table S1).

In terms of visualization, TimeTreeAnno is currently designed as a lightweight and efficient tool for fundamental annotations of time‐scaled trees. Its core functionality focuses on displaying geological time scales, precise node ages, HPD intervals, user‐defined text labels, and others. For more complex annotations, such as mapping trait data or evolutionary events, we recommend exporting the tree to specialized platforms like iTOL or ChiPlot [14, 29].

## CONCLUSIONS

To create a comprehensive, user‐friendly molecular dating suite, we have integrated the MCMCtree and r8s programs into PhyloSuite v2 and developed three new tools: MDGUI for molecular dating analysis, TimeTreeAnno for time‐scaled tree visualization, and MCMCTracer for convergence assessment of MCMC results. This suite retains the full functional repertoire of the original programs while introducing multiple important optimizations and novel features. These advancements include visual calibration configuration, support for common alignment formats, and automatic evolutionary model selection and implementation. The latter is significantly enhanced by integrating IQ‐TREE for Hessian matrix calculation, extending support beyond the native models of MCMCtree. Additional features, such as pausing/resuming, multithreading acceleration, and on‐demand MCMC summarization with convergence verification further streamline the analytical workflow. Beyond molecular dating, PhyloSuite v2 incorporates other major enhancements, such as sophisticated gene duplicate handling, accelerated data processing, deeper integration of the latest IQ‐TREE functions, and a refined adaptive interface for high‐resolution displays, alongside numerous bug fixes. Collectively, these developments further solidify the position of PhyloSuite as a seamless, end‐to‐end operational environment for evolutionary studies ‐ from data preparation and MCMC analysis to results visualization and diagnostic assessment. Its intuitive interface and streamlined workflow offer a significant improvement in usability and efficiency over existing tools. As an ongoing project, we welcome bug reports, feedback, and suggestions for future development and support of the research community.

## AUTHOR CONTRIBUTIONS

**Dong Zhao**: Writing—original draft; validation; visualization; software. **Tong Ye**: Validation; visualization; formal analysis; project administration; writing—original draft. **Fangluan Gao**: Conceptualization; writing—review and editing; methodology. **Ivan Jakovlić**: Writing—review and editing; validation; formal analysis; supervision. **Qiong La**: Methodology; supervision. **Yindong Tong**: Supervision; resources. **Xiang Liu**: Conceptualization; investigation; supervision. **Rui Song**: Conceptualization; investigation; supervision; resources. **Fei Liu**: Investigation; resources; supervision. **Zhong‐min Lian**: Investigation; supervision; resources. **Hong Zou**: Supervision; resources; investigation. **Wen‐Xiang Li**: Conceptualization; supervision; investigation. **Gui‐Tang Wang**: Conceptualization; investigation; supervision. **Benhe Zeng**: Conceptualization; investigation; supervision; resources. **Dong Zhang**: Software; writing—review and editing; funding acquisition; resources; methodology. All authors have read the final manuscript and approved it for publication.

## CONFLICT OF INTEREST STATEMENT

The authors declare no conflict of interest.

## ETHICS STATEMENT

No animals or humans were involved in this study.

## Supporting information


**Figure S1:** Comparison of time consumption of molecular dating analyses between the PhyloSuite v2 (4 threads) and standard MCMCtree (1 thread) (hour: minute).
**Figure S2:** Comparison of node divergence times and confidence intervals between the PhyloSuite v2 (4 threads) and standard MCMCtree (1 thread).
**Figure S3:** Comparison of time consumption for different functions between the old (v1) and new (v2) versions of PhyloSuite (minute: second).
**Figure S4:** The MCMCtree module interface automatically detects and displays the number of sequences and sequence type upon file import.
**Figure S5:** Fossil calibration interface showing the right‐click content menu operations.
**Figure S6:** Example of an annotated tree after adding the fossil calibration information.
**Figure S7:** MCMCTracer visualization displaying States, sampling frequency, ESS values, and diagnostic plots.
**Figure S8:** Convergence assessment module showing replicate analyses as dot plots comparing posterior means.
**Figure S9:** TimeTreeAnno visualization of “Figtree.tre” output, with selectable time units and geological timescales, showing confidence intervals and calibrated nodes.

**Table S1:** Troubleshooting: Some of the most common problems likely to be encountered by MDGUI users.

## Data Availability

The latest source code is available at https://github.com/dongzhang0725/PhyloSuite. Pre‐compiled versions for Windows, macOS, and Linux can also be downloaded from http://phylosuite.jushengwu.com/dongzhang0725.github.io/installation/#5-Download-links-for-users-in-China or https://github.com/dongzhang0725/PhyloSuite/releases. If the precompiled versions fail to run, you may try installing PhyloSuite via pip or conda. Detailed installation instructions are provided at http://phylosuite.jushengwu.com/dongzhang0725.github.io/installation/ or https://dongzhang0725.github.io/installation/. For detailed, step‐by‐step tutorials on performing molecular dating analyses in PhyloSuite v2, please refer to https://dongzhang0725.github.io/PhyloSuite‐demo/Molecular‐dating‐analysis/ or http://phylosuite.jushengwu.com/dongzhang0725.github.io/PhyloSuite‐demo/Molecular‐dating‐analysis/. Supplementary materials (figures, tables, graphical abstract, slides, videos, Chinese translated version, and updated materials) may be found in the online DOI or iMeta Science http://www.imeta.science/. The data that support the findings of this study are openly available in GitHub at https://dongzhang0725.github.io/.
