# Supplementary material for: PhyloSuite v2: The development of an all‐in‐one, efficient and visualization‐oriented suite for molecular dating analysis and other advanced features
Source: Imeta. 2025 Nov 25;4(6):e70095. doi: 10.1002/imt2.70095 (PMC12747537; doi:10.1002/imt2.70095)
Supplement: Supplementary file 1 — Figure S1: Comparison of time consumption of molecular dating analyses between the PhyloSuite v2 (4 threads) and standard MCMCtree (1 thread) (hour: minute). Figure S2: Comparison of node divergence times and confidence intervals between the PhyloSuite v2 (4 threads) and standard MCMCtree (1 thread). Figure S3: Comparison of time consumption for different functions between the old (v1) and new (v2) versions of PhyloSuite (minute: second). Figure S4: The MCMCtree module interface automatically detects and displays the number of sequences and sequence type upon file import. Figure S5: Fossil calibration interface showing the right‐click content menu operations. Figure S6: Example of an annotated tree after adding the fossil calibration information. Figure S7: MCMCTracer visualization displaying States, sampling frequency, ESS values, and diagnostic plots. Figure S8: Convergence assessment module showing replicate analyses as dot plots comparing posterior means. Figure S9: TimeTreeAnno visualization of “Figtree.tre” output, with selectable time units and geological timescales, showing confidence intervals and calibrated nodes. [file IMT2-4-e70095-s002.docx]

Supporting information to

**PhyloSuite v2: the development of an all-in-one, efficient and visualization-oriented suite for molecular dating analysis and other advanced features**

**Running title：**PhyloSuite v2 for molecular dating

Dong Zhao^1,2,3^, Tong Ye^1^, Fangluan Gao^4^, Ivan Jakovlić^1^, Qiong La^2^, Yindong Tong^5^, Xiang Liu^1^, , Rui Song^6^, Fei Liu^2,3^, Zhong-min Lian^1^, Hong Zou^7^, Wen-Xiang Li^7^, Gui-Tang Wang^7^, Benhe Zeng^3^*, Dong Zhang^1,2^*

^1^State Key Laboratory of Herbage Improvement and Grassland Agro-ecosystems, and College of Ecology, Lanzhou University, Lanzhou 730000, China

^2^Key Laboratory of Biodiversity and Environment on the Qinghai-Tibetan Plateau, Ministry of Education, School of Ecology and Environment, Xizang University, Lhasa 850032, China

^3^Institute of Fisheries Science, Xizang Academy of Agriculture and Animal Husbandry Sciences, Lhasa 850032, China

^4^Institute of Plant Virology, Fujian Agriculture and Forestry University, Fuzhou 350002, China

^5^School of Environmental Science and Engineering, Tianjin University, Tianjin 300072, China

^6^Hunan Fisheries Research Institute and Aquatic Products Seed Stock Station, Changsha 410153, China

^7^State Key Laboratory of Breeding Biotechnology and Sustainable Aquaculture, Institute of Hydrobiology, Chinese Academy of Sciences, Wuhan 430072, China

^#^These authors contributed equally: Dong Zhao, Tong Ye.

*Correspondence Dong Zhang, State Key Laboratory of Herbage Improvement and Grassland Agro-ecosystems, and College of Ecology, Lanzhou University, Lanzhou 730000, China. Email: dongzhang0725@gmail.com

Benhe Zeng, Institute of Fisheries Science, Xizang Academy of Agriculture and Animal Husbandry Sciences, Lhasa 850032, China. Email: [zengbh@xzaas.cn](mailto:zengbh@xzaas.cn)


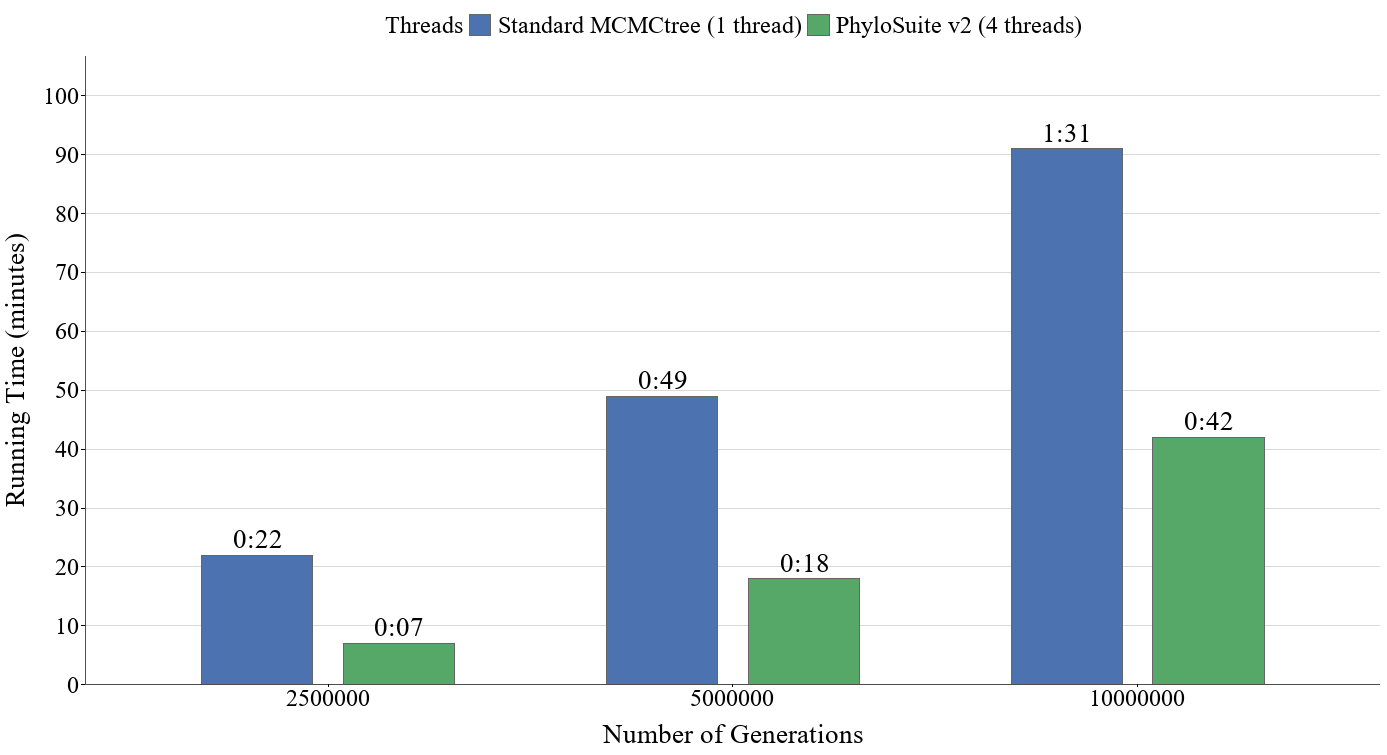


**Figure S1 Comparison of time consumption of molecular dating analyses between the PhyloSuite v2 (4 threads) and Standard MCMCtree (1 thread) (hour: minute).**


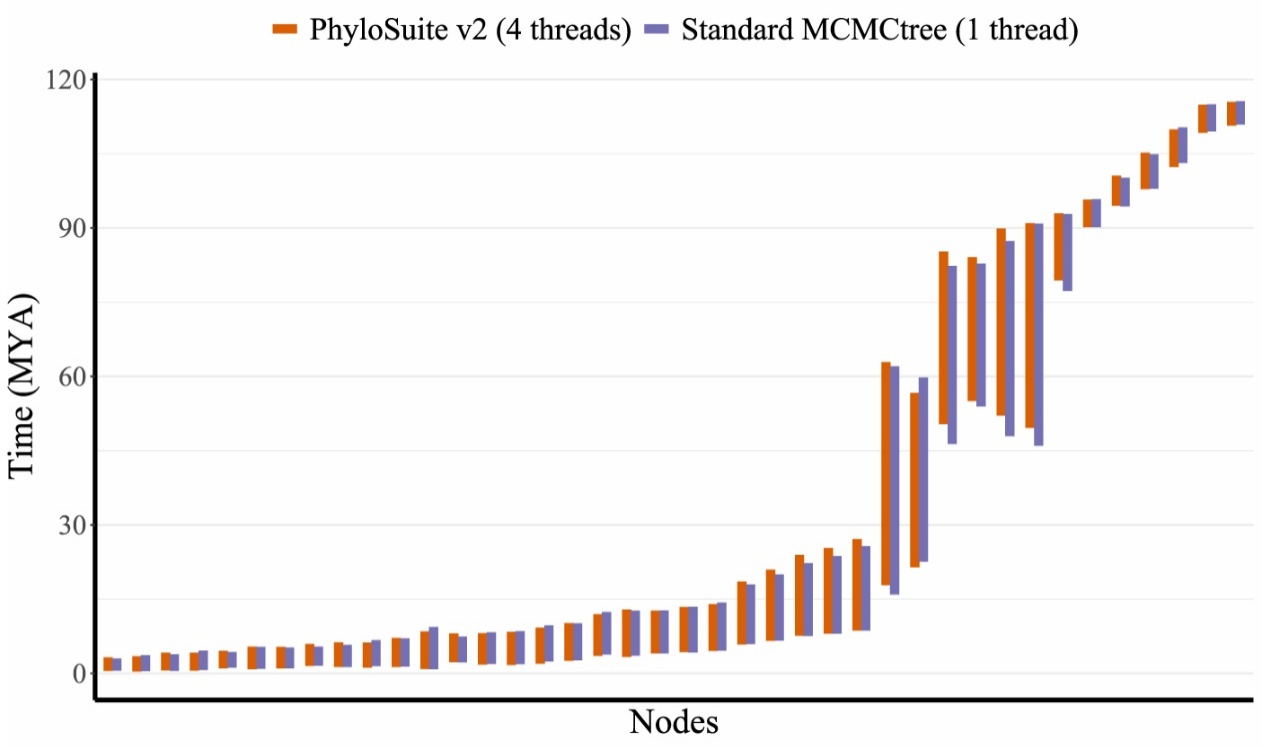


**Figure S2 Comparison of node divergence times and confidence intervals between the PhyloSuite v2 (4 threads) and Standard MCMCtree (1 thread).**


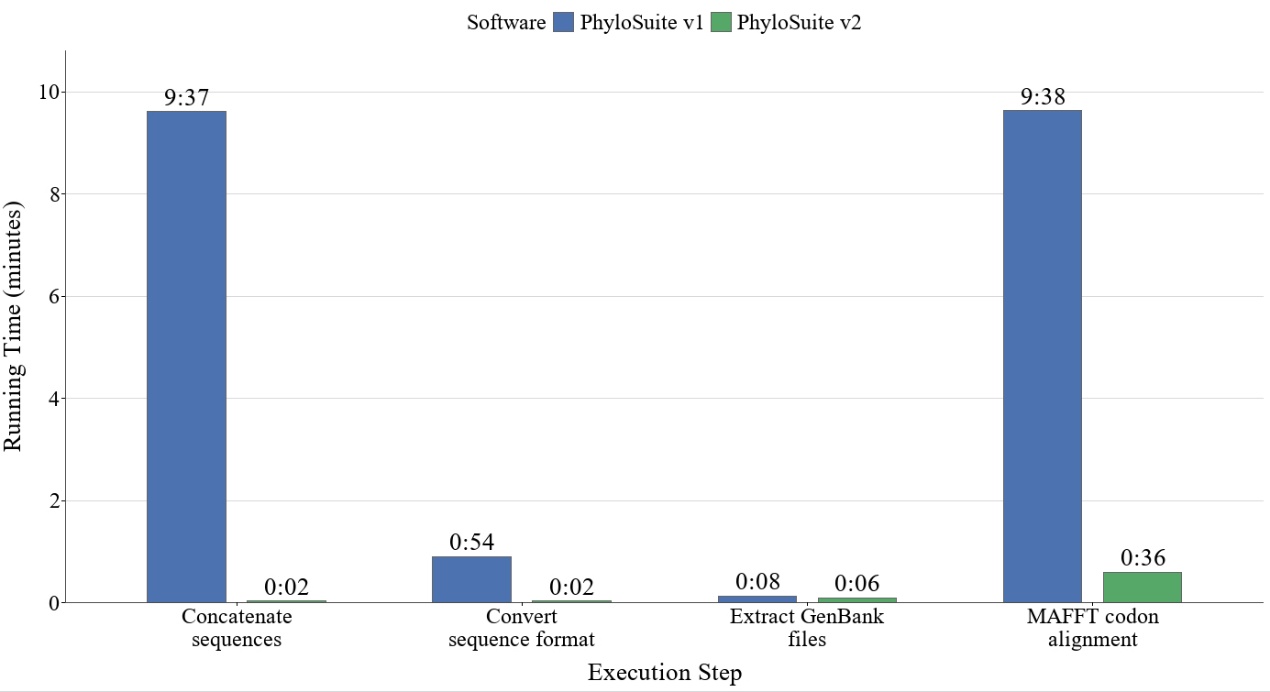


**Figure S3 Comparison of time consumption for different functions between the old (v1) and new (v2) versions of PhyloSuite (minute: second).**


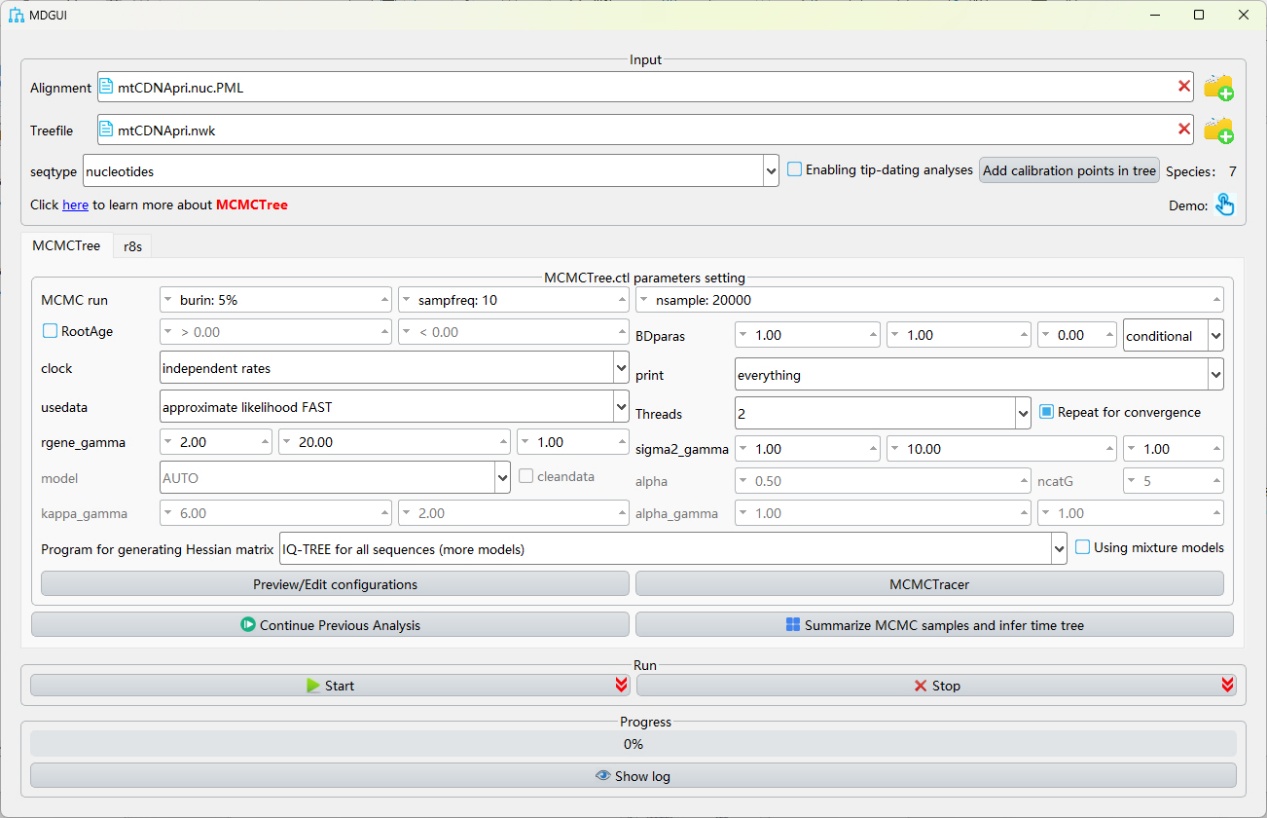


**Figure S4 The MCMCtree module interface automatically detects and displays the number of sequences and sequence type upon file import.** Users are required to manually configure other parameters.


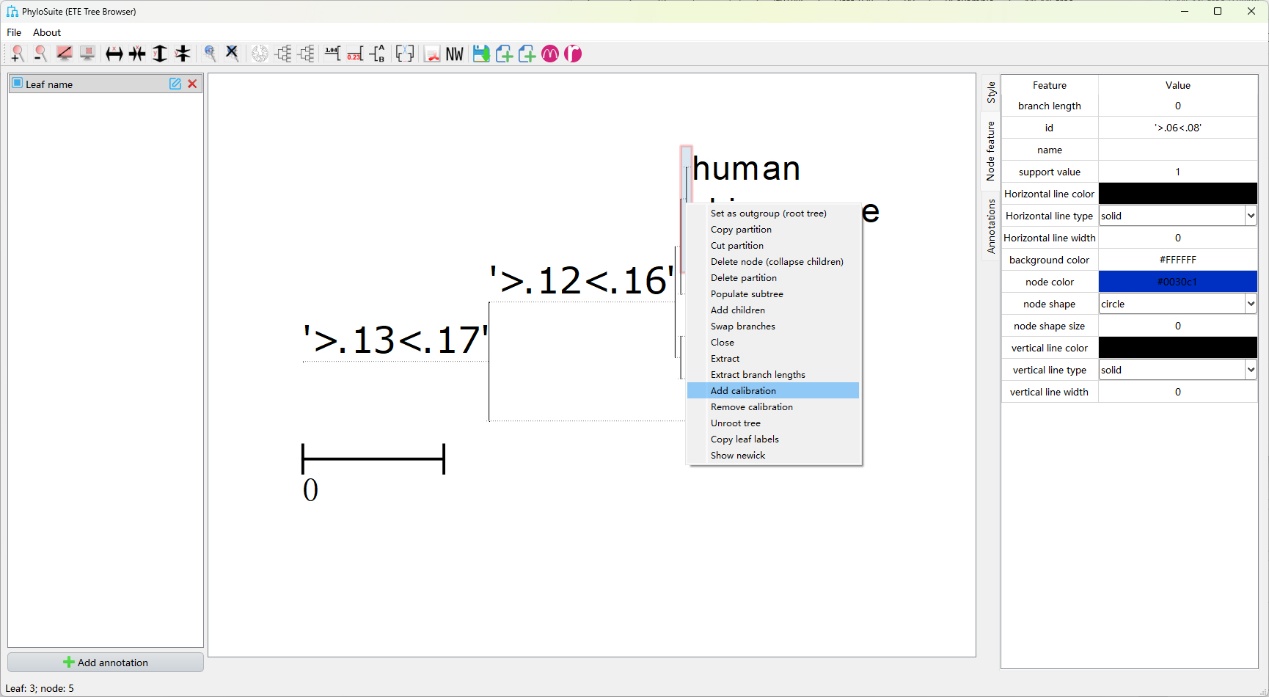


**Figure S5** **Fossil calibration interface showing the right-click context menu operations.** Users can click “Add calibration” button to add fossil calibration information to specific tree nodes.


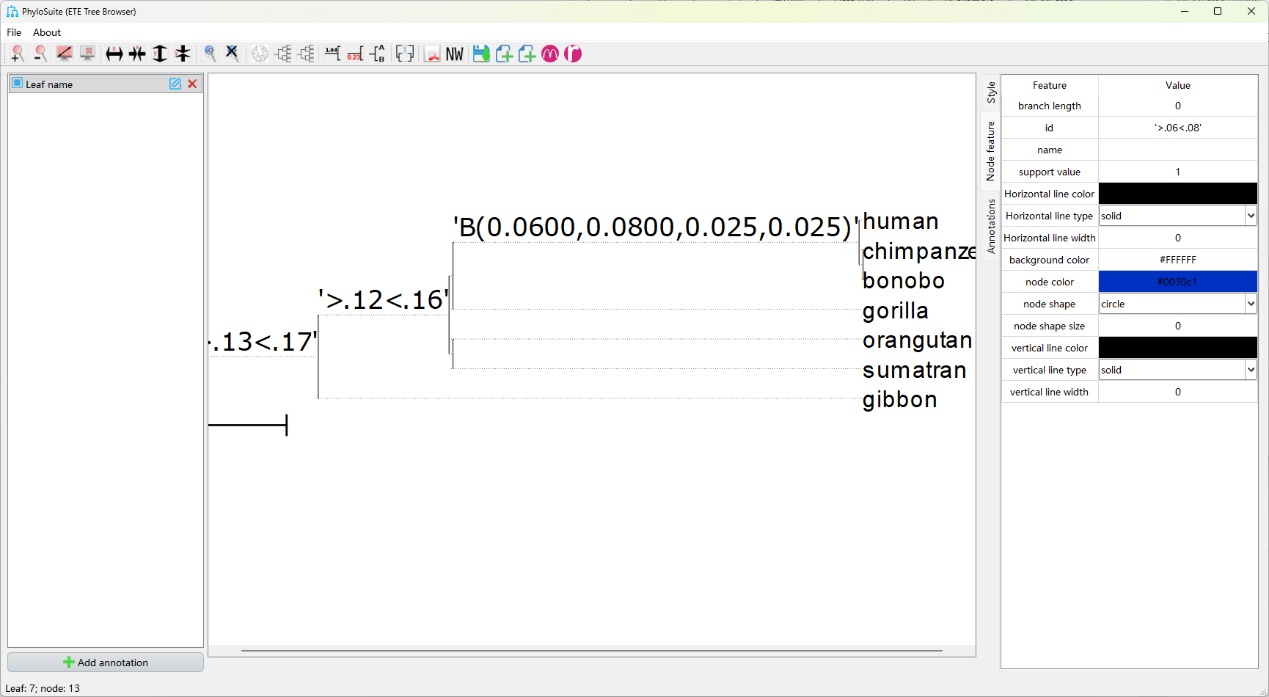


**Figure S6** **Example of an annotated tree after adding the fossil calibration information.** Click the “M” icon in the toolbar can save annotated trees.


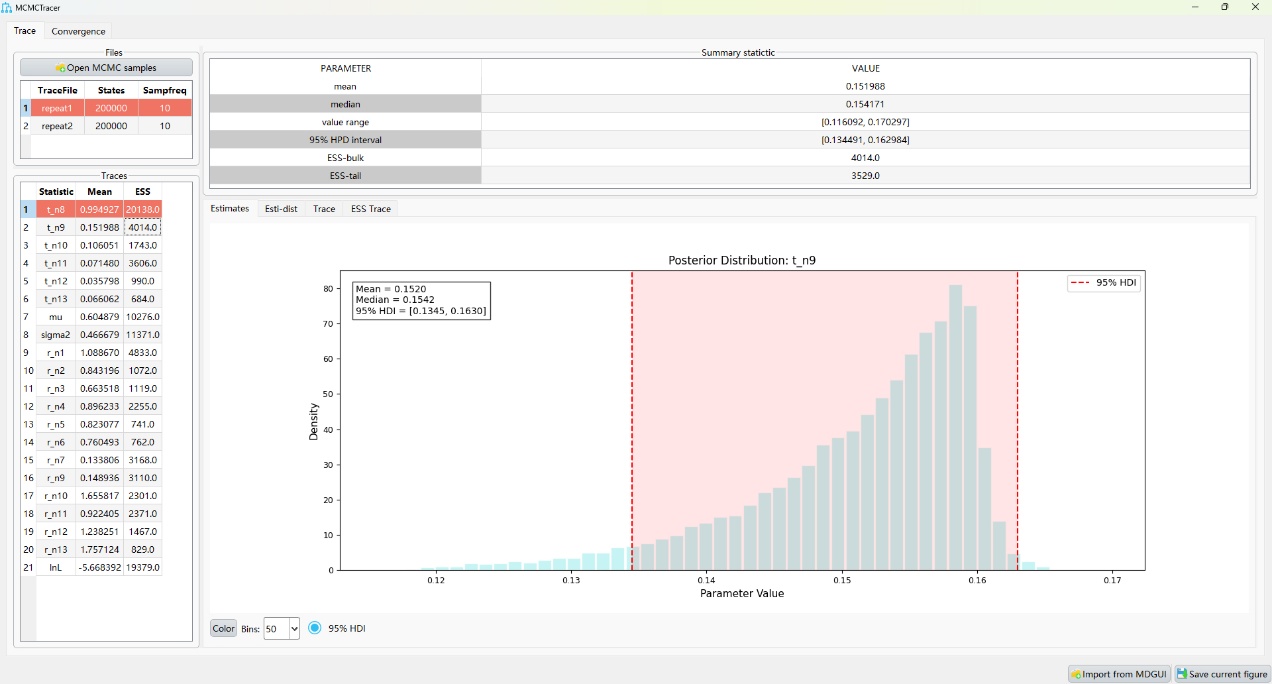


**Figure S7** **MCMCTracer visualization displaying States, sampling frequency, ESS values, and diagnostic plots.**


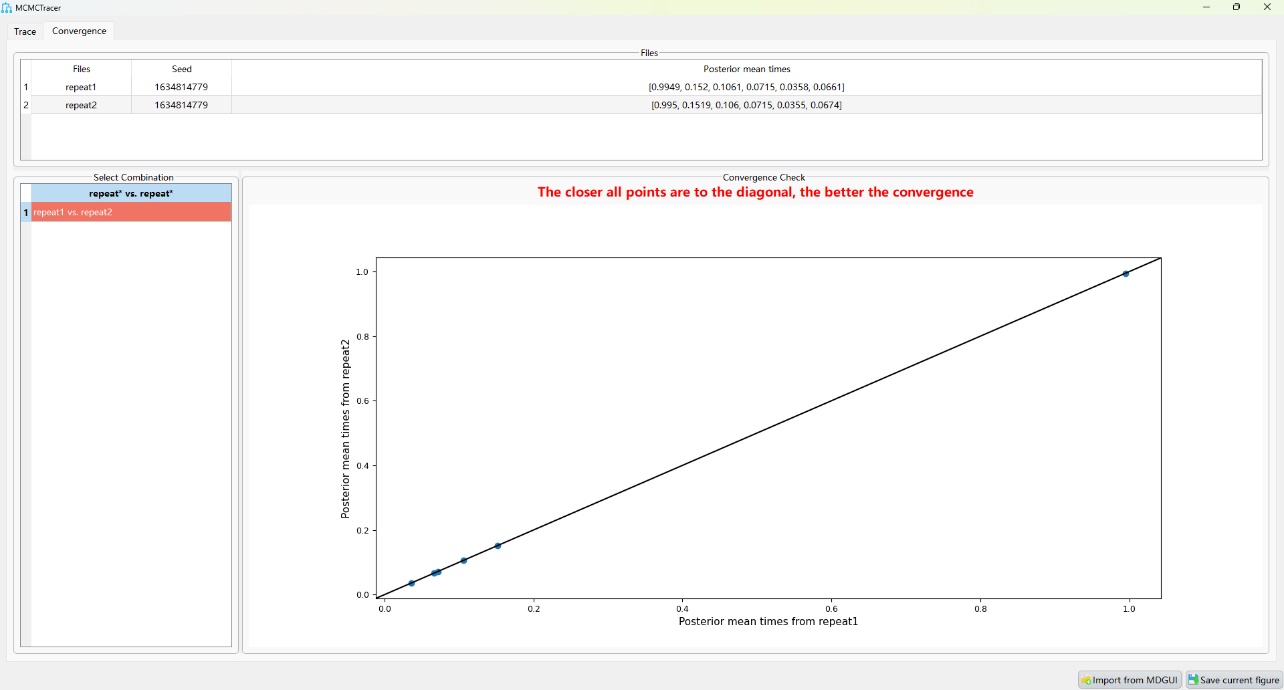


**Figure S8** **Convergence assessment module showing replicate analyses as dot plots comparing posterior means.**


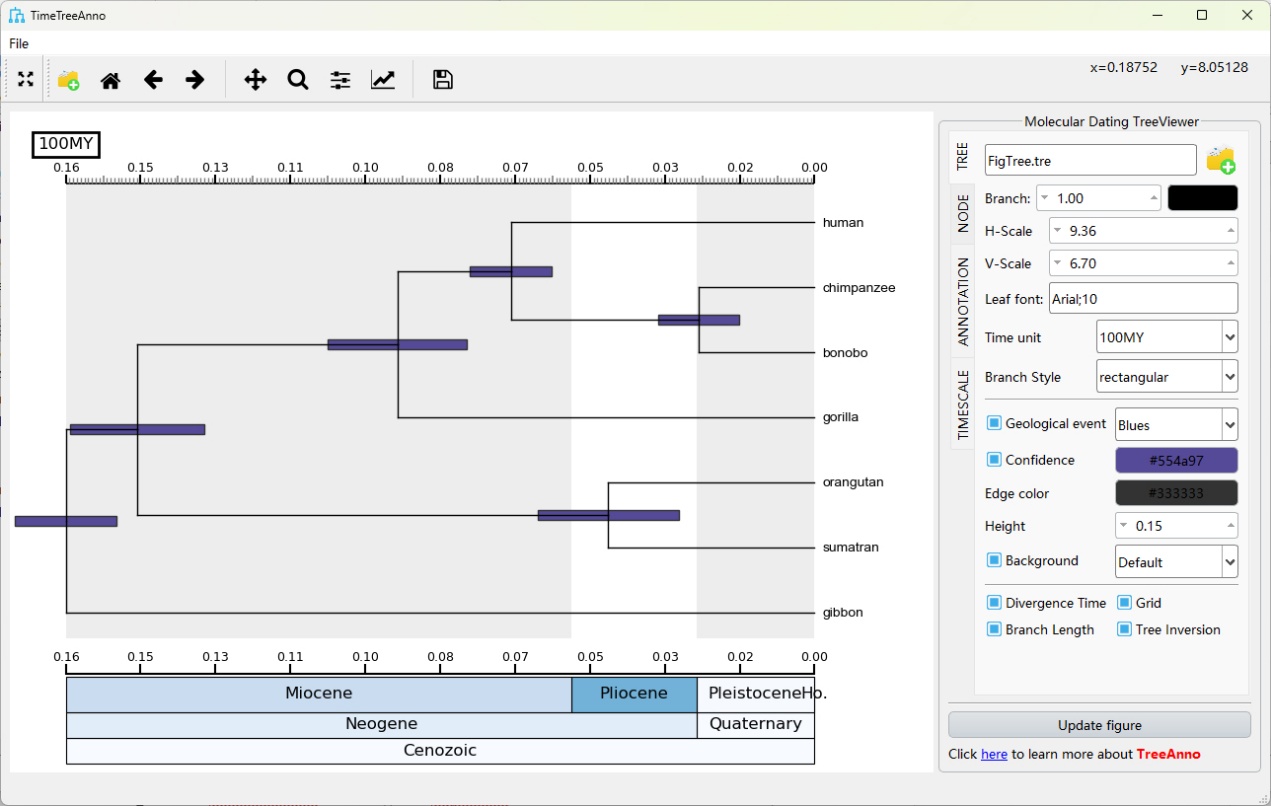


**Figure S9. TimeTreeAnno visualization of the “FigTree.tre” output, with selectable time units and geological timescales, showing confidence intervals and calibrated nodes.**
